# Personal exposure of PM_2.5_ and metabolic syndrome markers of pregnant women in South Korea: APPO study

**DOI:** 10.1007/s11356-023-30921-x

**Published:** 2023-11-23

**Authors:** Yeonseong Jeong, Sunwha Park, Eunjin Kwon, Young Min Hur, Young-Ah You, Soo Min Kim, Gain Lee, Kyung A. Lee, Soo Jung Kim, Geum Joon Cho, Min-Jeong Oh, Sung Hun Na, Se jin Lee, Jin-Gon Bae, Yu-Hwan Kim, Soo-Jeong Lee, Young-Han Kim, Young Ju Kim, The APPO study group

**Affiliations:** 1 Department of Obstetrics and Gynecology, Gangnam Severance Hospital, Yonsei University College of Medicine, Seoul, Korea; 2 Department of Obstetrics and Gynecology, College of Medicine, Ewha Medical Research Institute, Ewha Womans University Mokdong Hospital, Seoul, Korea; 3 Graduate Program in System Health Science and Engineering, Ewha Womans University, Seoul, Republic of Korea; 4 Department of Obstetrics and Gynecology, College of Medicine, Ewha Womans University Seoul Hospital, Seoul, Korea; 5 Department of Obstetrics and Gynecology, College of Medicine, Korea University Guro Hospital, Seoul, Korea; 6 Department of Obstetrics and Gynecology, College of Medicine, Kangwon National University Hospital, Chuncheon, Korea; 7 Department of Obstetrics and Gynecology, College of Medicine, Keimyung University Dongsan Medical Center, Daegu, Korea; 8 Department of Obstetrics and Gynecology, Ulsan university hospital, University of Ulsan College of Medicine Ulsan, Ulsan, Korea; 9 Department of Obstetrics and Gynecology, Institute of Women’s Life Medical Science, Yonsei University College of Medicine, Yonsei University Health System, Seoul, Republic of Korea

**Keywords:** Particulate matter, Indoor air pollution, Pregnancy complications, Metabolic dysfunction, Glucose intolerance, Lipid metabolism

## Abstract

We examined the association between exposure to PM_2.5_, focused on individual exposure level, and metabolic dysfunction during pregnancy. APPO study (Air Pollution on Pregnancy Outcome) was a prospective, multicenter, observational cohort study conducted from January 2021 to March 2023. Individual PM_2.5_ concentrations were calculated using a time-weighted average model. Metabolic dysfunction during pregnancy was assessed based on a modified definition of metabolic syndrome and its components, accounting for pregnancy-specific criteria. Exposure to PM_2.5_ during pregnancy was associated with worsened metabolic parameters especially glucose metabolism. In comparison to participants exposed to the low PM_2.5_ group, those exposed to high PM_2.5_ levels exhibited increased odds of gestational diabetes mellitus (GDM) after adjusting for confounding variables in different adjusted models. Specifically, in model 1, the adjusted odds ratio (aOR) was 3.117 with a 95% confidence interval (CI) of 1.234–7.870; in model 2, the aOR was 3.855 with a 95% CI of 1.255–11.844; in model 3, the aOR was 3.404 with a 95% CI of 1.206–9.607; and in model 4, the aOR was 2.741 with a 95% CI of 0.712–10.547. Exposure to higher levels of PM_2.5_ during pregnancy was associated with a tendency to worsen metabolic dysfunction markers specifically in glucose homeostasis. Further research is needed to investigate the mechanisms underlying the effects of ambient PM_2.5_ on metabolic dysfunction during pregnancy.

**Supplementary Information:**

The online version contains supplementary material available at 10.1007/s11356-023-30921-x.

## Background

Fine particulate matter 2.5 (PM_2.5_), consisting of particles with aerodynamic diameters ≤ 2.5 *μ*m, has exhibited a global increase in concentration despite efforts to reduce air pollution resulting from industrial development (Wallwork et al. 2017). Exposure to PM_2.5_ can result in respiratory ailments, cardiovascular complications, and impaired pulmonary function. Individuals at heightened vulnerability, such as pediatric and geriatric populations, have increased susceptibilities (Liu et al. 2019). In fact, the World Health Organization (WHO) has recognized air pollution as one of the top 10 global health hazards in 2019 (Wojtyla et al. 2020). Given the amplification of urbanization and industrial undertakings, comprehending the health ramifications of PM_2.5_ holds crucial significance for the formulation of efficacious strategies in public health management.

Previous studies have established the association between particulate matter and adverse pregnancy and neonatal outcomes, as pregnant women and fetuses are vulnerable to the effects of air pollution (Bai et al. 2020; Eze et al. 2015; Jirtle and Skinner 2007; Klepac et al. 2018; Lee et al. 2019; Li et al. 2021). However, the specific window of harmful exposure to PM_2.5_ during pregnancy remains inconclusive (Zhang et al. 2022).

Metabolic syndrome, characterized by central obesity, glucose intolerance, hypertriglyceridemia, and low high-density lipoprotein cholesterol (HDL-c), poses a significant public health concern and is associated with various complications, including chronic diseases such as cerebrovascular disease, diabetes mellitus, and cancer. The prevalence of metabolic syndrome in pregnancy has been increasing in tandem with advanced maternal age (Li et al. 2013). Previous animal studies have suggested that particulate matter induces oxidative stress and systemic inflammation, leading to metabolic dysfunction such as elevated blood pressure and altered lipid and glucose metabolism (Li et al. 2013). Similar findings have been reported in general populations (Brook and Rajagopalan 2009; Cheng et al. 2022).

Accurate individual measurement of particulate matter is crucial to comprehensively explore its effects. However, most studies investigating PM_2.5_ have been retrospective due to limitations in measurement techniques. Notably, particulate matter emissions originate not only from outdoor sources but also from indoor sources. Previous studies examining the association between particulate matter and pregnancy have primarily focused on outdoor particulate matter density, with limited evaluation of indoor particulate matter (Cheng et al. 2022; Jedrychowski et al. 2009; Shezi et al. 2020). Given that modern individuals, including pregnant women who may have reduced physical activity, spend a significant amount of time indoors, indoor particulate matter density should be considered when analyzing the impact of particulate matter pollution. Thus, the objective of this study was to investigate the effect of PM_2.5_ exposure on metabolic dysfunction in pregnant women using a personalized measurement approach.

## Methods

### Study design and sample collection

APPO study (Air Pollution on Pregnancy Outcome) was a prospective, multicenter, and observational cohort study conducted from January 2021 to March 2023. A total of 456 pregnant individuals were enrolled from the outpatient clinics of all participating institutions (Ewha Womans University Mokdong Hospital, Yonsei University Severance Hospital, Ewha Womans University Seoul Hospital, Korea University Guro Hospital, Kangwon National University Hospital, Keimyung University Dongsan Medical Center, and Ulsan University Hospital). Inclusion criteria for the study were: mothers aged 19 years or older, less than 28 weeks of singleton gestation, and no history of chronic medical illness, other gynecologic disease, and cancer. The identification of preexisting medical conditions was ascertained via either self-report or clinical assessment.

**Fig. 1:**
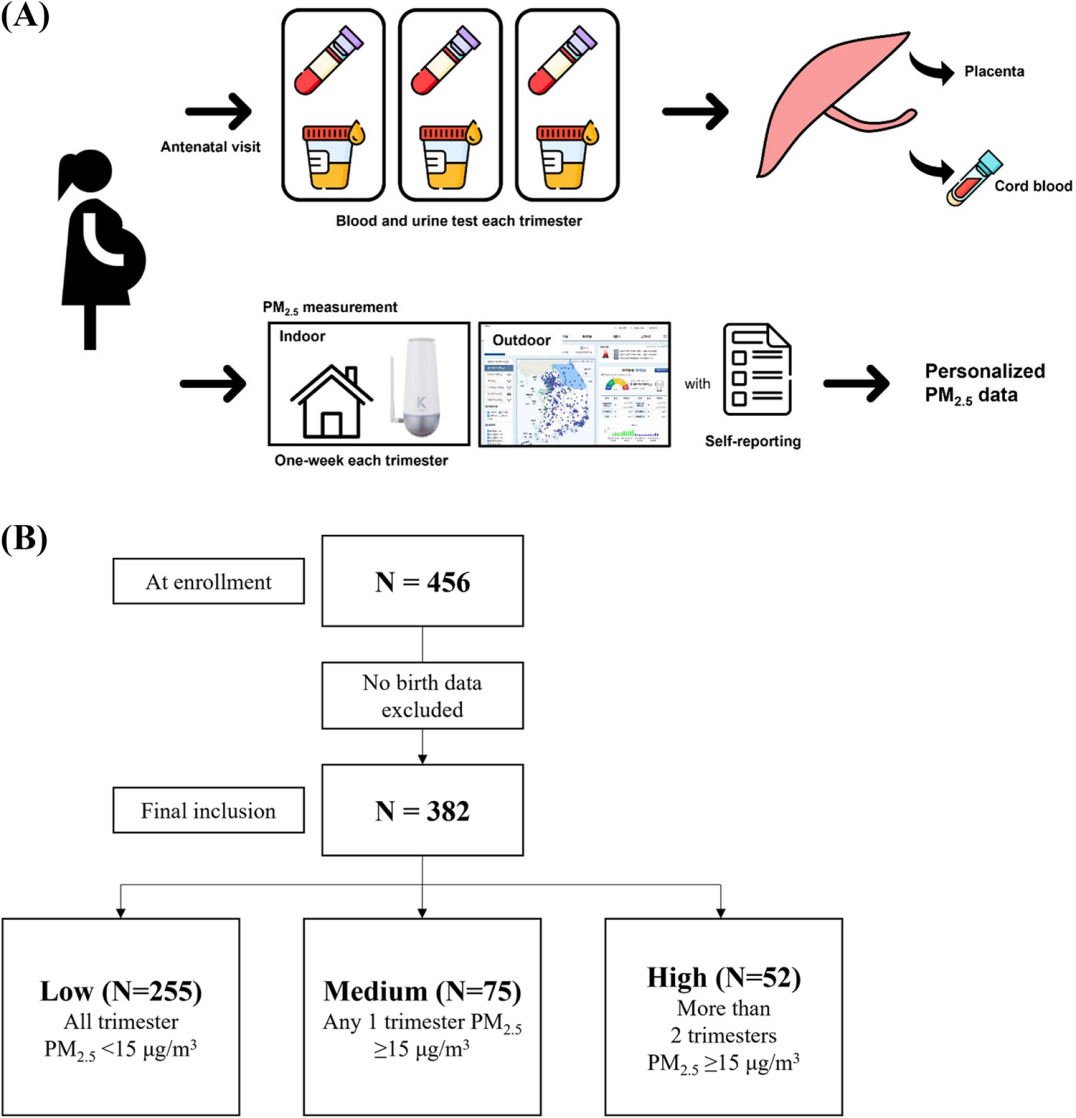
**A** Participant recruitment and sample collection processes. PM_2.5_, particulate matter 2.5. **B** Study flowchart

Sample collection involved obtaining blood and urine specimens from the pregnant women during routine antenatal visits at each trimester of pregnancy. Blood and urine specimens were collected in quantities of no less than 10 ml each. In addition, placenta and umbilical cord blood samples were collected at the time of delivery. The recruitment of participants and the sample collection processes are depicted in Fig. 1 for better understanding.

During the study period, self-reporting was conducted to collect data on various factors related to pregnant women, including age, pre-pregnancy body mass index (BMI), residential region, marriage status, education status, occupation, monthly household income level, gravidity (number of pregnancies), artificial reproductive technique, passive smoking exposure, drinking habits, exercise levels, physical activity levels, cooking device and technique, and air cleaner use (Hur et al. 2023).

### Measurement of particulate matter exposure

**Fig. 2:**
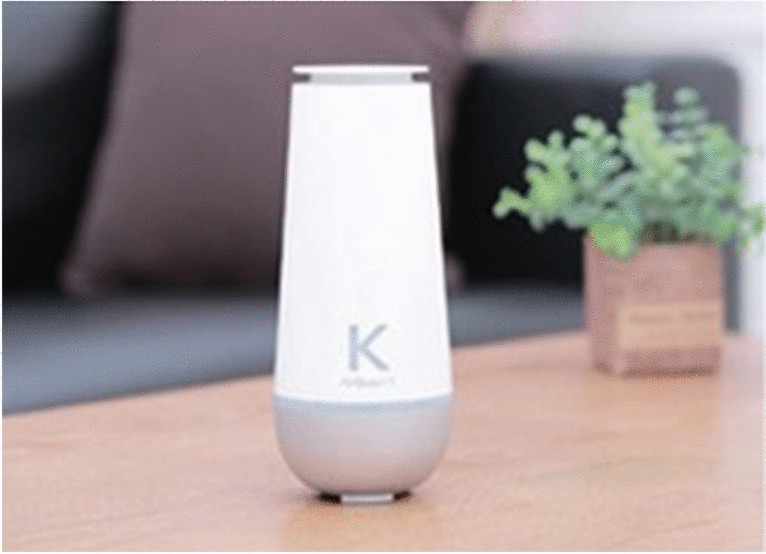
AirguardK® (Kweather, Co, Korea); a device for measurement of air pollutants, placed inside the participants’ house at least 1 week per trimester

AirguardK® (Kweather, Co, Korea) is a small electronic device equipped with a light scattering laser photometer sensor that is capable of detecting levels of air pollution. The device was placed inside the participants’ homes for a minimum of 1 week during each trimester of pregnancy to measure indoor air quality. The device collected data on various air pollutants such as PM_2.5_, PM_10_, and CO_2_, as well as temperature, humidity, and indoor integrated index, with a data collection interval of 1 min. The collected indoor air quality data was transmitted to an indoor air quality monitoring platform through Long-Term Evolution (LTE) communication network to prevent data loss, and data was collected and stored every minute. The measurement was carried out in real-time using Internet of Things (IoT) and Information and Communication Technology (ICT) for a week during each trimester of pregnancy to obtain concentration values of pollutants (Fig. 2).

Outdoor air pollutant concentrations were collected from a nearby urban atmospheric measurement network based on the addresses of the recruited pregnant women. The Urban Air Monitoring Station data used in this study were obtained from AirKorea (https://www.airkorea.or.kr/web) by The Korean Ministry of Environment. AirKorea has 614 stations in 162 cities and counties for measurement of air pollutant including sulfur dioxide (SO_2_), nitrogen dioxide (NO_2_), ozone (O_3_), carbon monoxide (CO), PM_10_, and PM_2.5_ as of year in Korea.

To assess the potential harmful effects of particulate matter, we evaluated human exposure levels, time-activity patterns, and indoor pollution levels. Participants completed a time-activity questionnaire for one week to capture their daily activities and locations, and outdoor PM exposure data was obtained from Air Korea and a mapping/application tool. We used a time-weighted average model to estimate individual PM exposure, which takes into account the duration and location of various activities, as described in previous studies (Edwards et al. 2001; Moschandreas et al. 2002; Park et al. 2020). This approach allows us to better understand the relationship between PM exposure and metabolic dysfunction during pregnancy by obtaining more accurate measurements of individual PM exposure through a time-activity pattern analysis.

$${C}_{per}=\sum ({T}_{indoor}\times {C}_{indoor}+{T}_{outdoor}\times {C}_{outdoor})$$

where

*C*_*per*_: predicted personal exposure
*T*_*indoor*_: indoor activity time rate (indoor activity time/24 h)
*C*_*indoor*_: indoor PM_2.5_ concentration
*T*_*outdoor*_: outdoor activity time rate (outdoor activity time/24 h)
*C*_*outdoor*_: outdoor PM_2.5_ concentration

According to the World Health Organization's (WHO) air quality guideline, a concentration of PM_2.5_ below 15 *μ*g per cubic meter (*μ*g/m^3^) is considered as the acceptable air quality level. In our study, we categorized the participants into three groups based on their PM_2.5_ exposure levels during pregnancy: low group: participants who were exposed to PM_2.5_ levels below 15 *μ*g/m^3^ throughout the entire pregnancy; medium group: participants who were exposed to PM_2.5_ levels equal to or greater than 15 *μ*g/m^3^ during any trimester of pregnancy; high group: participants who were exposed to PM_2.5_ levels above 15 *μ*g/m^3^ more than two trimesters in pregnancy (Organization 2021). This categorization was done to assess the potential association between PM_2.5_ exposure levels and metabolic dysfunction during pregnancy in a more systematic and standardized manner.

### Definition of metabolic dysfunction in pregnancy

Based on the criteria for metabolic syndrome outlined by the International Diabetes Federation, metabolic syndrome was defined as a waist circumference of ≥ 35 inches for women, along with abnormal metabolic components such as elevated blood pressure, altered lipid metabolism, and glucose intolerance. However, we found it inappropriate to apply the original diagnostic criteria for metabolic syndrome in our study due to the unique metabolic changes that occur during pregnancy. Additionally, there were inconsistencies in the trimester of pregnancy during which measurements were taken, and not all pregnant women underwent testing in a fasting state. Therefore, we modified the definition of metabolic parameters in our study by gestational diabetes mellitus (GDM) diagnosis, as a measure of fasting glucose levels. Additionally, we modified the lipid profile criteria to include triglyceride (TG) levels ≥ 175 mg/dl and high-density lipoprotein (HDL) levels < 40 mg/dl, based on previous research (Nordestgaard et al. 2016; Rammah et al. 2021). These modifications were made to account for the unique metabolic changes that occur during pregnancy and to ensure the appropriateness and accuracy of our analysis with regard to metabolic dysfunction. If a participant met the criteria for two or more metabolic parameters (elevated blood pressure over 130/85 mmHg, diagnosis of GDM, TG ≥ 175 mg/dl, and HDL < 40 mg/dl), it was considered indicative of metabolic dysfunction.

To diagnose GDM, following the rule of each institution, all pregnant women underwent the GDM screening tests between 24 and 28 weeks of gestation. GDM was diagnosed when thresholds were met or exceeded (Cunningham et al. 2022). If there is any abnormal value in OGTT, it is defined as glucose intolerance.

Lipid profiles including LDL cholesterol, triglycerides, and total cholesterol were measured through maternal blood samples in the third trimester. HDL cholesterol (HDL-c) was calculated using the traditional Friedewald equation (Friedewald et al. 1972). It has been known that elevated triglycerides (TG) are a risk factor for insulin resistance while an increase in HDL-c is a protective factor, especially since TG/HDL-c ratio ≥ 3.0 mg/dl has a higher association with insulin resistance than TG and HDL itself. Thus, we evaluated the TG/HDL-c ratio as a marker of metabolic dysfunction in this study (Barat et al. 2018; Gong et al. 2021; Marotta et al. 2010).

### Statistical analysis

Categorical variables were expressed by frequencies out of available cases. Quantitative variables were expressed as mean (standard deviation), median and ranges. Differences in the continuous and categorical variables were analyzed using Student *t*-test, ANOVA, and chi-square test or Fisher’s exact test, as appropriate. The chi-square test for linear-by-linear association was used for analysis of trends. The logistic regression models were used to analyze the associations between exposure to PM_2.5_ and metabolic dysfunction using odds ratio (OR) and 95% confidence intervals (CI). Covariates, which are variables that may confound the relationship between the exposure and outcome, were selected based on previous research and included maternal age, monthly household income, education levels, pre-pregnancy BMI, exercise levels, physical activity levels, passive smoking exposure, season of conception (spring [March to May], summer [June to August], autumn [September to November], winter [December to February]), cooking device, cooking technique, and air cleaner use (Eze et al. 2015; Zhang et al. 2020). We examined the association by 4 steps: a crude (unadjusted) model 1; model 2, adjusted for demographical factors (age, education, income), pre-pregnancy BMI, and season of conception; model 3, adjusted for lifestyle factors such as smoking history, alcohol habit, physical activity levels, exercise, cooking method, and air cleaner use; and model 4, adjusted for environment factors (VOC, CO2, Temp, humidity, and PM_10_). The dose–response curves were drawn using Probit analysis. Receiver operating curves (ROC) and the area under the curve (AUC) were used to analysis. The exposure effects were regarded as statistically significant at the *p* < 0.05 level. Data was analyzed using SPSS (IBM) software version 26.0.

## Results

### Demographics

During the study period, we included 382 participants who had measured PM_2.5_ concentrations for more than two trimesters and had complete birth data out of a total of 456 participants (Fig. 1B). The baseline characteristics of the study population, such as maternal age, marital status, education level, smoking status, and alcohol consumption, are presented in Table 1. The mean maternal age was 33.59 ± 4.09 years, and most participants were married, well-educated (91.4% completed university education), non-smokers, and non-alcohol consumers.

**Table 1:** Demographic characteristics and clinical data of the participants at baseline visit (*N* = 382)

Characteristics	Total (*n* = 382)	PM_2.5_ exposure levels			
		Low (*n* = 255)	Medium (*n* = 75)	High (*n* = 52)	*p*-value
Age (year)	33.49 ± 4.06	33.59 ± 3.71	33.36 ± 4.68	33.23 ± 4.75	0.834
Pre-pregnancy BMI (kg/m^2^)	21.58 ± 3.25	21.69 ± 3.46	21.26 ± 2.99	21.49 ± 2.47	0.590
Pre-pregnancy BMI over 25 kg/m^2^	44 (11.5)	30 (11.8)	10 (13.3)	4 (7.7)	0.605
Region (*n*, %)					0.895
Urban	358 (93.7)	236 (92.5)	72 (96.0)	50 (96.2)	
Suburban	16 (4.2)	12 (4.7)	3 (4.0)	1 (1.9)	
Mountainous area	2 (0.5)	2 (0.8)	0 (0.0)	0 (0.0)	
Industrial area	6 (1.6)	5 (2.0)	0 (0.0)	1 (1.9)	
Marital status (*n*, %)					0.131
Married	377 (98.7)	253 (99.2)	74 (98.7)	50 (96.2)	
Unmarried, divorced, diseased	5 (1.3)	2 (0.8)	1 (1.3)	2 (3.8)	
Education status (*n*, %)					0.208
Lower than high school	18 (4.8)	15 (5.9)	2 (2.7)	1 (1.9)	
High school	15 (3.9)	6 (2.4)	4 (5.3)	5 (9.6)	
College or university	272 (71.2)	182 (71.4)	57 (76.0)	33 (63.5)	
Postgraduate	77 (20.2)	52 (20.4)	12 (16.0)	13 (25.0)	
Occupation status (*n*, %)					0.887
No	119 (31.2)	83 (32.5)	25 (33.3)	11 (21.2)	
Yes	263 (68.8)	172 (67.5)	50 (66.7)	41 (78.8)	
Monthly income level (*n*, %)					0.734
< KRW 400 (× 10^4^)	94 (24.6)	54 (26.5)	25 (36.8)	15 (32.5)	
KRW 400 ~ 600 (× 10^4^)	67 (17.5)	43 (21.1)	13 (19.1)	11 (23.9)	
≥ KRW 600 (× 10^4^)	112 (16.0)	75 (36.7)	21 (30.9)	16 (34.8)	
Gravidity (*n*, %)					0.765
Primigravida	188 (49.2)	122 (47.8)	37 (49.3)	29 (55.8)	
Multigravida	194 (50.8)	133 (52.2)	38 (50.7)	23 (44.2)	
Pregnancy route (*n*, %)					0.676
Natural	325 (85.1)	220 (86.3)	61 (81.3)	44 (84.6)	
ART (IUI, IVF-ET)	57 (14.9)	35 (13.7)	14 (18.7)	8 (15.4)	
Smoking status (*n*, %)					0.736
Former	348 (91.1)	232 (91.0)	70 (93.3)	46 (88.5)	
Never	34 (8.9)	23 (9.0)	5 (6.6)	6 (11.3)	
Passive smoking in house (*n*, %)					0.119
Yes	21 (5.5)	10 (3.9)	6 (8.0)	5 (9.6)	
No	361 (94.5)	245 (96.1)	69 (92.0)	47 (90.4)	
Passive smoking in work (*n*, %)					0.918
Yes	30 (7.9)	21 (8.2)	6 (8.0)	3 (5.9)	
No	351 (91.9)	234 (91.8)	69 (92.0)	48 (94.1)	
Drinking status (days/week) (*n*, %)					0.525
Former	269 (70.4)	169 (82.8)	58 (85.3)	42 (89.4)	
Never	50 (13.1)	35 (17.2)	10 (14.7)	5 (10.6)	
Exercise (*n*, %)					0.114
Yes	78 (20.4)	198 (78.0)	66 (88.0)	39 (75.0)	
No	303 (79.3)	56 (22.0)	9 (12.0)	13 (25.0)	
Physical activity (*n*, %)					0.445
Active	9 (2.4)	7 (2.8)	1 (1.3)	1 (2.0)	
Moderate	86 (22.5)	60 (23.6)	16 (21.3)	10 (19.6)	
Inactive	285 (74.6)	187 (73.6)	58 (77.3)	40 (78.4)	
Cooking device (*n*, %)					0.738
Stove	192 (50.3)	119 (51.7)	46 (63.0)	27 (52.9)	
Induction	134 (35.1)	91 (39.6)	23 (31.5)	20 (39.2)	
Etc	28 (7.4)	20 (8.7)	4 (5.5)	4 (7.8)	
Cooking technique (*n*, %)					0.533
Fry/grill/roast	282 (73.8)	186 (78.8)	61 (85.9)	35 (72.9)	
Steam/boil	44 (11.5)	33 (14.0)	5 (7.0)	6 (12.5)	
Raw	6 (1.6)	4 (1.7)	1 (1.4)	1 (2.1)	
Etc	23 (6.0)	13 (5.5)	4 (5.6)	6 (12.5)	
Season of conception (*n*, %)					NA
Spring (March to May)	114 (29.8)	99 (39.0)	10 (13.3)	5 (9.6)	
Summer (June to August)	115 (30.1)	40 (15.7)	42 (56.0)	33 (63.5)	
Autumn (September to November)	88 (23.0)	59 (23.2)	18 (24.0)	11 (21.2)	
Winter (December to February)	64 (16.8)	56 (22.0)	5 (6.7)	3 (5.8)	

Data are presented as *N* (%) or mean ± SD

Low group: participants who were exposed to PM_2.5_ levels below 15 *μ*g/m^3^ throughout the entire pregnancy. Medium group: participants who were exposed to PM_2.5_ levels equal to or greater than 15 *μ*g/m^3^ during any trimester of pregnancy. High group: participants who were exposed to PM_2.5_ levels above 15 *μ*g/m^3^ more than two trimester in pregnancy

*BMI* body mass index, *KRW* Korean won, *ART* artificial reproductive technique, *IUI* intrauterine insemination, *IVF-ET* in vitro fertilization-embryo transfer

NA (not applicable) as statistically not interpretable

### PM_2.5_ distribution and environment factors of study population

The distribution of PM_2.5_ concentrations and other air conditions are described in Table 2. The median concentration of PM_2.5_ weighted for time-activity log was 9.89 *μ*g/m^3^ (IQR 6.35–16.02) over the entire follow-up period. The median indoor PM_2.5_ exposure level was 9.52 *μ*g/m^3^ (IQR 5.85–15.75) and outdoor PM_2.5_ exposure was 17.51 *μ*g/m^3^ (IQR 14.28–21.75). During the study period, median daily temperature was 25.10 °C (IQR 23.89–26.43) and relative humidity was 37.99% (IQR 31.96–43.55).

**Table 2:** Descriptive statistics of air pollutants in study period

Variables	Mean (SD)	Min	Q1	Median	Q3	Max
Air pollutant concentration						
PM_2.5_ (*μ*g/m^3^)						
Indoor	12.41 (10.60)	0.90	5.85	9.52	15.75	99.05
Outdoor	18.14 (5.96)	6.00	14.28	17.51	21.75	38.95
Weighted PM_2.5_^#^	12.71 (10.19)	1.04	6.35	9.89	16.02	99.05
PM_10_ (*μ*g/m^3^)	23.08 (19.81)	2.49	11.96	17.48	26.46	199.73
CO_2_ (ppm)	873.64 (327.40)	277.12	651.74	833.20	1019.61	2820.20
VOC (*μ*g/m^3^)	5069.34 (5306.09)	31.26	1332.73	2960.65	6681.92	28276.41
Weather conditions						
Daily temperature (℃)	25.22 (2.25)	17.45	23.89	25.10	26.428	34.75
Relative humidity (%)	38.61 (9.51)	17.04	31.96	37.99	43.551	84.00

*VOC* volatile organic compounds, *Min* minimum, *Q1* 25 quartile, *Q3* 75 quartile, *Max* maximum

^#^Weighted PM_2.5_ *C*_*per*_= ∑( *T*_*indoor*_ × *C*_*indoor*_ + *T*_*outdoor*_ × *C*_*outdoor*_); where *C*_*per*_: predicted personal exposure, *T*_*indoor*_: indoor activity time rate (indoor activity time/24 h), *C*_*indoor*_: indoor PM_2.5_ concentration, *T*_*outdoor*_: outdoor activity time rate (outdoor activity time/24 h); *C*_*outdoor*_: outdoor PM_2.5_ concentration

### PM_2.5_ exposure and metabolic dysfunction in pregnancy

We observed significant correlations between PM_2.5_ exposure and metabolic components, such as elevated blood pressure, glucose intolerance (defined as any one abnormal oral glucose tolerance test (OGTT) results), gestational diabetes mellitus (GDM), and altered lipid metabolism as shown in Table 3. The longer the duration of exposure to high PM_2.5_ concentrations, the higher the risk of glucose intolerance and GDM (*p* = 0.023, 0.045, respectively). There was also a trend of increasing levels of triglycerides over 175 mg/dl with prolonged exposure to high PM_2.5_ concentrations (*p* for trend 0.055).

**Table 3:** Association between each PM_2.5_ levels and metabolic components

Variables	PM_2.5_ exposure levels				
	Low (*n* = 255)	Medium (*n* = 75)	High (*n* = 52)	*p*-value	*p* for trend
HBP (SBP ≥ 130 mmHg or DBP ≥ 85 mmHg)	6 (2.4)	7 (9.5)	1 (2.0)	0.020*	0.345
Glucose intolerance	12 (4.7)	6 (8.0)	8 (15.4)	0.023*	0.006*
GDM	14 (5.5)	7 (9.3)	8 (15.4)	0.045*	0.012*
HDL-c < 40 mg/dl	27 (19.4)	9 (21.4)	1 (4.2)	0.157	0.171
TG ≥ 175 mg/dl	211 (93.8)	63 (96.9)	45 (100.0)	0.187	0.055
TG/HDL ≥ 3.0	119 (85.6)	31 (73.8)	19 (79.2)	0.178	0.170
Metabolic dysfunction	53 (20.8)	19 (25.3)	14 (26.9)	0.507	0.147

Data are presented as *N* (%)

Analysis was conducted using the chi-square test, Fisher’s exact test, and the linear-by-linear association. Increased exposure to PM impacts glucose metabolism and may also influence elevated blood pressure and triglyceride levels, although the significance of these effects is limited or statistically insignificant

Low group: participants who were exposed to PM_2.5_ levels below 15 /m^3^ throughout the entire pregnancy. Medium group: participants who were exposed to PM_2.5_ levels equal to or greater than 15 *μ*g/m^3^ during any trimester of pregnancy. High group: participants who were exposed to PM_2.5_ levels above 15 *μ*g/m^3^ more than two trimesters in pregnancy

*HBP* high blood pressure, *GDM* gestational diabetes, *HDL-c* high density lipoprotein cholesterol, *TG* triglycerides

*p-value < 0.05

**Fig. 3:**
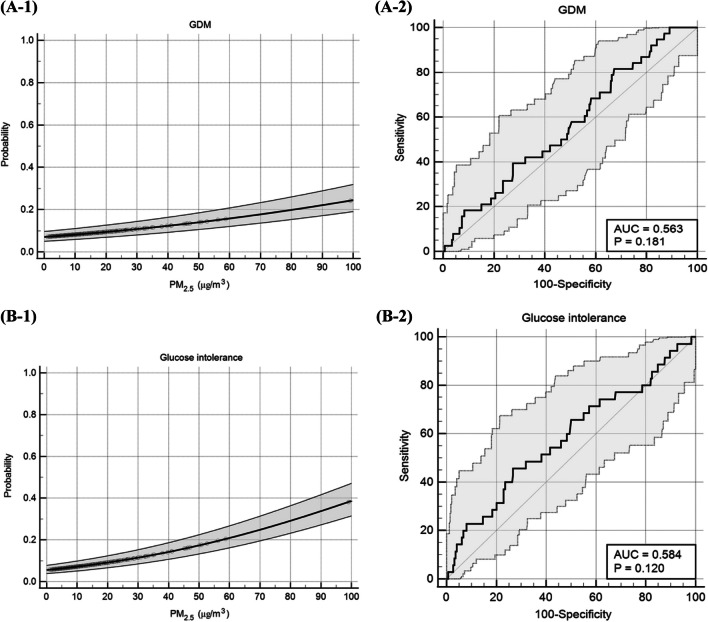
Concentration–response relationship and receiver operating curve (ROC) between exposure of PM_2.5_ and glucose metabolism. **A** GDM (1) concentration–response curve and (2) ROC curve; **B** Glucose intolerance (1) concentration–response curve and (2) ROC curve

Table 4 presents the odds ratio (OR) of metabolic components by PM_2.5_ exposure period during pregnancy, after adjusting for four types of models; demographic, lifestyle, and environmental factors (as described in the “Methods” section). As the exposure to higher concentrations of PM_2.5_ for an extended period increased, changes in glucose homeostasis observed. Glucose intolerance showed an odds ratio (OR) of 3.682 (95% CI 1.423–9.525, *p* = 0.007) in the crude regression model for the high group, and similar results were obtained when considering other covariates. In the case of gestational diabetes mellitus (GDM), the crude model for the High group showed an OR of 3.117 (95% CI 1.234–7.870, *p* = 0.016), and even in models considering other variables, generally consistent results were observed. The highest exposure to PM_2.5_ in most trimesters was found to be associated with GDM, as evidenced by the concentration–response curves shown in Fig. 3A-1 and ROC curve in Fig. 3A-2 (AUC 0.563, *p* = 0.161). Exposure to high PM_2.5_ concentrations was also associated with glucose intolerance after adjustments, as shown by the concentration–response curve in Fig. 3B-1 and ROC curve in Fig. 3B-2 (AUC 0.584, *p* = 0.130).

**Table 4:** Adjusted odds ratios with 95% CI of metabolic dysfunction and its components in overall population with increase PM_2.5_ exposure levels

		Low (ref)	Medium		High	
			OR (95% CI)	*p*-value	OR (95% CI)	*p*-value
HBP	Model 1	1	4.318 (1.404–13.279)	0.011*	0.827 (0.097–7.017)	0.827
	Model 2		4.038 (0.948–17.192)	0.059	0.703 (0.065–7.592)	0.703
	Model 3		4.536 (1.379–14.920)	0.013*	0.810 (0.092–7.128)	0.810
	Model 4		6.484 (0.813–51.725)	0.078	NA	
Glucose intolerance	Model 1	1	1.761 (0.638–4.863)	0.275	3.682 (1.423–9.525)	0.007*
	Model 2		2.593 (0.722–9.321)	0.144	5.792 (1.681–19.961)	0.050*
	Model 3		2.222 (0.681–7.248)	0.186	5.026 (1.669–15.137)	0.004*
	Model 4		2.272 (0.477–10.829)	0.303	5.001 (1.228–20.359)	0.025*
GDM	Model 1	1	1.765 (0.685–4.547)	0.239	3.117 (1.234–7.870)	0.016*
	Model 2		1.654 (0.544–5.025)	0.375	3.855 (1.255–11.844)	0.018*
	Model 3		2.180 (0.742–6.402)	0.156	3.404 (1.206–9.607)	0.021*
	Model 4		2.142 (0.542–8.467)	0.277	2.741 (0.712–10.547)	0.142
HDL-c < 40	Model 1	1	1.131 (0.484–2.643)	0.776	0.180 (0.023–1.395)	0.101
	Model 2		1.161 (0.430–3.136)	0.769	0.185 (0.022–1.572)	0.185
	Model 3		0.974 (0.387–2.454)	0.956	0.195 (0.024–1.554)	0.195
	Model 4		2.059 (0.634–6.692)	0.230	0.316 (0.035–2.852)	0.305
TG ≥ 175	Model 1	1	2.090 (0.463–9.443)	0.338	NA	
	Model 2		1.635 (0.340–7.870)	0.540	NA	
	Model 3		1.329 (0.271–6.5110	0.726	NA	
	Model 4		1.565 (0.156–15.685)	0.703	NA	
Metabolic dysfunction	Model 1	1	1.293 (0.708–2.361)	0.403	1.404 (0.709–2.781)	0.330
	Model 2		1.305 (0.672–2.535)	0.432	1.215 (0.556–2.656)	0.625
	Model 3		1.247 (0.653–2.381)	0.503	1.265 (0.600–2.667)	0.537
	Model 4		1.294 (0.540–3.103)	0.563	0.779 (0.256–2.376)	0.661

Analysis was conducted using the logistic regression. Increased exposure to PM impacts glucose metabolism and may also influence elevated blood pressure and triglyceride levels, although the significance of these effects is limited or statistically insignificant

*HBP* high blood pressure, *GDM* gestational diabetes, *HDL-c* high density lipoprotein cholesterol, *TG* triglycerides, *T-chol* total cholesterol

Low group: participants who were exposed to PM_2.5_ levels below 15 *μ*g/m^3^ throughout the entire pregnancy. Medium group: participants who were exposed to PM_2.5_ levels equal to or greater than 15 *μ*g/m^3^ during any trimester of pregnancy. High group: participants who were exposed to PM_2.5_ levels above 15 *μ*g/m^3^ more than two trimesters in pregnancy

Model 1, a crude (unadjusted) model; model 2, adjusted for demographical factors (age, education, income), pre-pregnancy BMI, and season of conception; model 3, adjusted for lifestyle factors such as smoking history, alcohol habit, physical activity levels, exercise, cooking method, and air cleaner use; and model 4, adjusted for environment factors (VOC, CO_2_, temperature, humidity, and PM_10_)

NA (not applicable): the effect size was judged to be clinically not interpretable

*p-value < 0.05

**Fig. 4:**
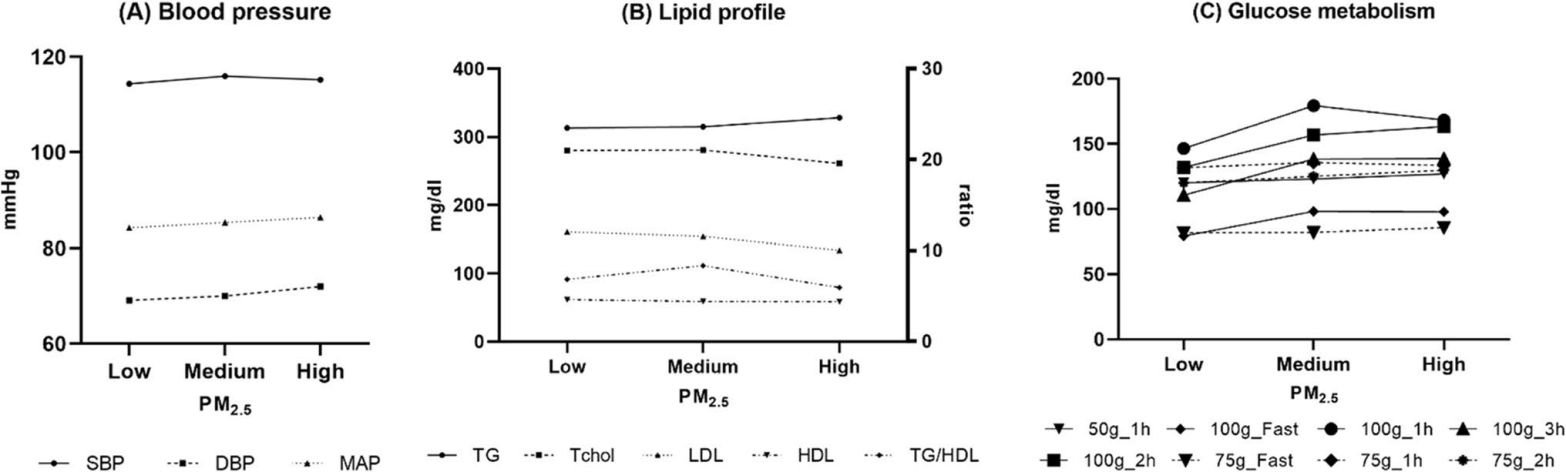
Trend of metabolic components according to PM_2.5_ exposure level. **A** Blood pressure in 3rd trimester, **B** lipid profile, and (**C**) glucose metabolism. These graphs represent the average values of metabolic components for each group. In **A**, there was an increase in DBP and MAP, but no significant change in SBP. In **B**, a trend of increased TG was observed. Furthermore, **C** an increasing tendency in fasting glucose, post-2 h glucose after a 100-g OGTT, and post-1 h glucose after a 50-g OGTT. Abbreviations: SBP, systolic blood pressure; DBP, diastolic blood pressure; MAP, mean arterial pressure; TG, triglycerides; T-chol, total cholesterol; LDL, low density lipoprotein; HDL, high density lipoprotein; Fast, fasting

Figure 4 illustrates the mean values of metabolic parameters corresponding to each cohort. Figure 4A reveals an elevation in diastolic blood pressure (DBP) and mean arterial pressure (MAP), albeit lacking a noteworthy alteration in systolic blood pressure (SBP). In Fig. 4B, a discernible trend towards increased triglyceride (TG) levels is evident. Furthermore, Fig. 4C demonstrates a gradual incline in fasting glucose levels, glucose levels at the 2-h mark following a 100-g oral glucose tolerance test (OGTT), and glucose levels at the 1-h mark following a 50-g OGTT.

### Sensitivity analysis

When sensitivity analysis was performed on pregnant individuals with a pre-pregnancy BMI less than 27 kg/m^3^, sustained exposure to PM_2.5_ demonstrated an association with elevated blood pressure in the third trimester and impaired glucose metabolism (Table S1). Additionally, an observable trend of triglyceride increase suggests that PM_2.5_ exposure might impact lipid metabolism. The association between exposure to PM_2.5_ during pregnancy and metabolic components is presented in Table S2.

## Discussion

This study aimed to investigate the association between particulate matter (PM) and metabolic dysfunction during pregnancy by utilizing personalized measurements of PM_2.5_ concentration. The findings of this study revealed a positive association between PM_2.5_ exposure during pregnancy and metabolic dysfunction. Specifically, it was observed that PM_2.5_ exposure during pregnancy was associated with alterations in blood pressure control, lipid metabolism, and glucose homeostasis. Pregnant women who were exposed to high concentrations of PM_2.5_ exhibited elevated blood pressure in their third trimester, increased levels of triglycerides (TGs), and higher odds of gestational diabetes mellitus (GDM) and glucose intolerance.

Particulate matter (PM) is derived from various sources, such as industrial emissions, vehicular emissions, and wildfires, with approximately 25% of PM attributed to combustion of fuel. However, during pregnancy, reduced mobility or increased prevalence of remote work may result in increased indoor activities. As a consequence, it is imperative to consider indoor sources of PM when analyzing its effects on pregnancy outcomes. Indoor sources of PM can have a significant impact on air quality and exposure during pregnancy. Indoor exposure to PM can be influenced by multiple factors such as ventilation, cleaning, cooking, heating, tobacco smoke, building materials, and individual behaviors, and may vary depending on geographical location, cultural practices, and other contextual factors. This approach can provide a comprehensive understanding of the potential risks and impacts of PM on maternal and fetal health during pregnancy. Therefore, incorporating the consideration of indoor sources of PM in the analysis and interpretation of research findings can enhance the accuracy of assessing the potential health effects of PM during pregnancy (Ambade et al. 2022; Kumar et al. 2020; Wojtyla et al. 2020; Zhang et al. 2022).

Several studies have provided evidence that is consistent with the findings of this study, indicating that exposure to PM_2.5_ is associated with metabolic dysfunction. Specifically, higher levels of PM_2.5_ have been associated with metabolic syndrome, hypertriglyceridemia, and elevated fasting blood glucose levels (Eze et al. 2015; Liu et al. 2022; Wallwork et al. 2017). Recent studies have also reported an association between PM_2.5_ exposure and gestational diabetes mellitus (GDM) (Bai et al. 2020; Zhang et al. 2020). Barat et al. demonstrated that long-term exposure to particulate matter is a risk factor for dyslipidemia and elevated TG/HDL ratio, and may even be associated with GDM (Barat et al. 2018; Mao et al. 2020).

In contrast, while the majority of previous studies have shown a positive association between particulate matter and metabolic dysfunction, some studies have reported negative or null associations. Honda et al. suggested that exposure to PM_2.5_ during the pre-conception period and first trimester did not increase the risk of gestational diabetes mellitus (GDM). Furthermore, some studies have reported no association between PM_2.5_ exposure and glucose intolerance, but have observed an increase in HbA1c levels with higher PM_2.5_ concentrations (Honda et al. 2017; Li et al. 2021; Lucht et al. 2018; Robledo et al. 2015). It is hypothesized that the lack of prospective studies may contribute to these discrepancies.

The mechanistic association between fine particulate matter exposure and metabolic disorders, in accordance with prior research, is summarized as follows. Exposure to PM_2.5_ triggers systemic inflammation and pulmonary oxidative stress. Elevated concentrations of inflammatory mediators such as tumor necrosis factor-α (TNF- α) and interleukin-6 (IL-6), pivotal cytokines in the inflammatory cascade, lead to an intensified inflammatory response. This heightened response could potentially induce endothelial cell impairment, precipitating insulin resistance. Furthermore, via fluid shifts to the third space, this detriment may also contribute to the initiation of preeclamptic conditions (Ambade et al. 2022; Baron et al. 1995; Herder et al. 2016; Kumar et al. 2020; Sun et al. 2009). Further research is needed to elucidate the mechanisms underlying the relationship between particulate matter exposure during pregnancy and adverse fetal outcomes.

The potential role of oxidative stress as a mechanistic pathway linking particulate matter exposure to metabolic dysfunction during pregnancy has been hypothesized and investigated in recent research. Oxidative stress refers to an imbalance between the production of reactive oxygen species (ROS) and the body’s capacity to counteract and repair ROS-induced damage. Various sources of oxidative stress, such as surrounding metals, tobacco, airborne particulate matter, and plastics, have been implicated in including systemic inflammation and disrupting metabolic pathways, ultimately leading to metabolic syndrome and insulin resistance (Ruano et al. 2022). The inhalation of particulate matter has been demonstrated to induce oxidative stress and trigger systemic inflammation, vascular dysfunction, atherosclerosis, and cardiovascular disease in non-pregnant populations (Dabass et al. 2016; Ruano et al. 2022). Moreover, ROS and oxygen-centered free radicals generated by particulate matter have been shown to impair the insulin signaling pathway, resulting in insulin resistance (Alderete et al. 2017; Araujo and Nel 2009; Brook et al. 2010; Houstis et al. 2006; Hutcheson and Rocic 2012; Münzel et al. 2017; Perticone et al. 2001; Roeckner et al. 2016). Furthermore, oxidative stress induced by particulate matter has been associated with various adverse obstetric outcomes, including recurrent pregnancy loss, prematurity, intrauterine growth restriction (IUGR), diabetes, and preeclampsia (Oke and Hardy 2021; Ornoy et al. 2021; van Westering-Kroon et al. 2021; Yu et al. 2020; Zejnullahu et al. 2021). To mitigate the detrimental effects of oxidative stress on inflammation and metabolic dysfunction, antioxidants have been investigated as a potential intervention (Casuso and Huertas 2018; Gregório et al. 2016; L.S et al. 2016). However, it should be noted that the use of antioxidants as a preventive or therapeutic strategy for particulate matter-induced metabolic dysfunction during pregnancy is still an area of ongoing research, and further studies are needed to comprehensively evaluate their efficacy, safety, and optimal dosages. Additionally, addressing the underlying causes of particulate matter pollution and implementing measures to reduce exposure to particulate matter remain critical in safeguarding maternal and fetal health during pregnancy.

Investigating the impact of particulate matter on adverse pregnancy outcomes in crucial in the context of fetal programming, as external stimuli experienced by the fetus in the uterus, such as oxidative stress, maternal physiological changes, and preeclampsia, can have long-term effects on fetal health (Hur et al. 2022; Kwon and Kim 2017; Öztürk and Türker 2021). Previous retrospective studies have suggested that particulate matter exposure during pregnancy may lead to fetal neurodevelopmental delay, possibly due to inflammation, although the underlying mechanisms are not fully understood (Lin et al. 2022; Su et al. 2022). These findings highlight that exposure to particulate matter during pregnancy may also influence maternal metabolic dysfunction. Consistent with the fetal programming theory, particulate matter exposure could alter the intrauterine environment, which plays a significant role in the fetal health beyond maternal well-being.

The principal strength of this study is its status as the first prospective investigation conducted in South Korea examining the impact of particulate matter on pregnant women. Additionally, this study concentrates on the individual concentration of particulate matter, which represents a unique contribution to the existing literature. Nevertheless, this study has limitations. Firstly, this study was limited by small sample size. We anticipate that more people will enroll in this study through ongoing investigations. Secondly, the study was conducted during the 2019 coronavirus pandemic, which may have resulted in reduced outdoor activities and may not fully represent outdoor PM_2.5_ concentrations. Thirdly, the lack of available data on PM_2.5_ exposure during the pre-conception period, which hinders the ability to assess the impact of PM_2.5_ prior to pregnancy. The omission of a measurement period is a potential limitation. Finally, it should be noted that the composition of PM_2.5_ can vary across different countries, and the present study did not assess the specific composition of PM_2.5_. This may have implications for the observed adverse health outcomes and represents a potential limitation of the study. However, this study was conducted during the period of the COVID-19 pandemic, thus indoor air quality measurements were exceedingly advantageous for the analysis. Even in the post-pandemic era, environmental pollution continues to intensify alongside global warming, and it is anticipated that indoor activities will correspondingly escalate. It is hoped that the findings of this study will be beneficial in the post-COVID era.

## Conclusion

The findings of this study revealed a positive association between PM_2.5_ exposure during pregnancy and metabolic dysfunction especially in glucose metabolism. These results emphasize the significance of managing PM_2.5_ concentrations to mitigate potential alterations in metabolic components that may contribute to adverse pregnancy outcomes. Furthermore, further research is warranted to investigate the effects of particulate matter on metabolic dysfunction during pregnancy in more depth.

### Supplementary Information

Below is the link to the electronic supplementary material.

Supplementary file1 (DOCX 24 KB)

## Data Availability

All data generated or analyzed during this study are included in this published article (and its additional information files).

## Abbreviations

*PM*_*2.5*_: Particulate matter 2.5
*sBP*: Systolic blood pressure
*dBP*: Diastolic blood pressure
*MAP*: Mean arterial pressure
*TG*: Triglycerides
*Tchol*: Total cholesterol
*LDL**-C*: Low density lipoprotein cholesterol
*HDL-C*: High density lipoprotein cholesterol
*SD*: Standard deviation
*aOR*: Adjusted odds ratio
*CI*: Confidence intervals
*GDM*: Gestational diabetes mellitus
*WHO*: World Health Organization
*SO*_*2*_: Sulfur dioxide
*CO*_*2*_: Carbon dioxide
*NO*_*2*_: Nitrogen dioxide
*CO*: Carbon monoxide
*LTE*: Long-Term Evolution
*BMI*: Body mass index
*OGTT*: Oral glucose tolerance test
*CRP*: C-reactive protein
*IUGR*: Intrauterine growth restriction
*TNF- α*: Tumor necrosis factor-α
*IL-6*: Interleukin-6
