# Supplementary material for: Personal exposure of PM2.5 and metabolic syndrome markers of pregnant women in South Korea: APPO study
Source: Environ Sci Pollut Res Int. 2023 Nov 23;30(59):123893–906. doi: 10.1007/s11356-023-30921-x (PMC10746774; doi:10.1007/s11356-023-30921-x)
Supplement: Supplementary file 1 — Supplementary file1 (DOCX 24 KB) [file 11356_2023_30921_MOESM1_ESM.docx]

**Table 1S. Association between each PM_2.5_ levels and metabolic components in maternal pre-pregnancy BMI <27kg/m^2^ (N=361).**

|  | **PM_2.5_ exposure levels** | |  |  |  |
| --- | --- | --- | --- | --- | --- |
| **Variables** | **Low (n=238)** | **Medium (n=72)** | **High (n=51)** | **p value** | **p for trend** |
| HBP (SBP ≥130 mmHg or DBP ≥85 mmHg) | 21 (8.8) | 8 (11.1) | 9 (18.0) | 0.156 | 0.046* |
| Glucose intolerance | 11 (4.6) | 6 (8.3) | 8 (15.7) | 0.019* | 0.006* |
| GDM | 12 (5.1) | 7 (9.7) | 7 (13.7) | 0.057 | 0.017* |
| HDL-c <40 mg/dl | 26 (19.3) | 7 (17.5) | 1 (4.3) | 0.250 | 0.071 |
| TG ≥175 mg/dl | 201 (93.5) | 60 (96.8) | 44 (100.0) | 0.187 | 0.028* |
| TG/HDL ≥3.0 | 116 (85.9) | 29 (72.5) | 18 (78.3) | 0.116 | 0.081 |
| Metabolic dysfunction | 50 (21.0) | 16 (22.2) | 13 (25.5) | 0.779 | 0.273 |

* p-value < 0.05

Data are presented as N (%)

Analysis was conducted using the Chi-square test, Fisher’s exact test, and the linear-by-linear association.

Low group: Participants who were exposed to PM_2.5_ levels below 15 µg/m^3^ throughout the entire pregnancy.; Medium group: Participants who were exposed to PM_2.5_ levels equal to or greater than 15 µg/m^3^ during any trimester of pregnancy.; High group: Participants who were exposed to PM_2.5_ levels above 15 µg/m^3^ more than two trimesters in pregnancy.

Abbreviations: HBP (high blood pressure), GDM (gestational diabetes), HDL-c (high density lipoprotein cholesterol), TG (triglycerides)

**Table 2S. Adjusted odds ratios with 95 % CI of metabolic dysfunction and its components in overall population with increase PM_2.5_ exposure levels in maternal pre-pregnancy BMI <27kg/m^2^ (N=361).**

|  |  | **Low (ref)** | **Medium** |  | **High** |  |
| --- | --- | --- | --- | --- | --- | --- |
|  |  |  | **OR (95% CI)** | **p-value** | **OR (95% CI)** | **p-value** |
| **HBP** | Model 1 | 1 | 1.292 (0,546-3.055) | 0.560 | 2.268 (0.970-5.303) | 0.059 |
|  | Model 2 |  | 1.241 (0.443-3.481) | 0.681 | 2.271 (0.775-6.656) | 0.135 |
|  | Model 3 |  | 1.151 (0.453-2.925) | 1.151 | 2.026 (0.808-5.083) | 0.132 |
|  | Model 4 |  | 1.421 (0.339-5.964) | 0.631 | 2.647 (0.760-9.219) | 0.126 |
| **Glucose intolerance** | Model 1 | 1 | 1.876 (0.669-5.264) | 0.232 | 3.839 (1.459-10.101) | 0.006* |
|  | Model 2 |  | 2.257 (0.568-8.969) | 0.247 | 6.297 (1.507-26.315) | 0.012* |
|  | Model 3 |  | 2.660 (0.787-8.989) | 0.115 | 5.698 (1.816-17.875) | 0.003* |
|  | Model 4 |  | 1.967 (0.401-9.664) | 0.405 | 3.675 (0.697-19.379) | 0.125 |
| **GDM** | Model 1 | 1 | 2.019 (0.764-5.338) | 0.157 | 2.983 (1.112-8.000) | 0.030* |
|  | Model 2 |  | 1.871 (0.611-5.724) | 0.272 | 3.066 (0.972-9.675) | 0.056 |
|  | Model 3 |  | 3.069 (0.980-9.606) | 0.054 | 3.647 (1.161-11.456) | 0.027* |
|  | Model 4 |  | 2.127 (0.539-8.389) | 0.281 | 2.323 (0.547-9.870) | 0.254 |
| **HDL-c <40** | Model 1 | 1 | 0.889 (0.354-2.234) | 0.803 | 0.191 (0.025-1.479) | 0.113 |
|  | Model 2 |  | 0.881 (0.299-2.595) | 0.818 | 0.201 (0.024-1.711) | 0.142 |
|  | Model 3 |  | 0.215 (0.026-1.811) | 0.215 | 0.689 (0.248-1.908) | 0.473 |
|  | Model 4 |  | 1.446 (0.408-5.118) | 0.568 | 0.376 (0.040-3.505) | 0.390 |
| **TG ≥175** | Model 1 | 1 | 2.090 (0.462-9.453) | 0.339 | NA |  |
|  | Model 2 |  | 1.611 (0.341-7.613) | 0.547 | NA |  |
|  | Model 3 |  | 1.359 (0.272-6.788) | 0.709 | NA |  |
|  | Model 4 |  | 0.517 (0.041-6.497) | 0.609 | NA |  |
| **Metabolic dysfunction** | Model 1 | 1 | 1.074 (0.568-2.032) | 0.826 | 1.286 (0.637-2.597) | 0.483 |
|  | Model 2 |  | 1.112 (0.513-2.411) | 0.789 | 1.124 (0.458-2.760) | 0.799 |
|  | Model 3 |  | 1.115 (0.554-2.245) | 0.760 | 1.249 (0.566-2.758) | 0.582 |
|  | Model 4 |  | 0.905 (0.353-2.321) | 0.835 | 0.666 (0.208-2.127) | 0.493 |

* p-value < 0.05

Abbreviations: HBP (high blood pressure), GDM (gestational diabetes), HDL-c (high density lipoprotein cholesterol), TG (triglycerides), T-chol (total cholesterol)

Low group: Participants who were exposed to PM_2.5_ levels below 15 µg/m^3^ throughout the entire pregnancy.; Medium group: Participants who were exposed to PM_2.5_ levels equal to or greater than 15 µg/m^3^ during any trimester of pregnancy.; High group: Participants who were exposed to PM_2.5_ levels above 15 µg/m^3^ more than two trimesters in pregnancy.

Model 1, a crude (unadjusted) model; model 2, adjusted for demographical factors (age, education, income), pre-pregnancy BMI, season of conception; model 3, adjusted for lifestyle factors such as smoking history, alcohol habit, physical activity levels, exercise, cooking method, air cleaner use; and model 4, adjusted for environment factors (VOC, CO_2_, temperature, humidity, and PM_10_)

NA (not applicable): The effect size was judged to be clinically not interpretable
